# Universal Adhesive Bonding to Sound and Artificially Demineralized Dentin: The Role of Etching Strategy and Resin Composite Type

**DOI:** 10.3390/ma19194227

**Published:** 2026-10-05

**Authors:** Fehime Alkan Aygör, Hasibe Sevilay Bahadır, Özlem Seçkin Kelten

**Affiliations:** Department of Restorative Dentistry, Faculty of Dentistry, Ankara Yildirim Beyazit University, Ankara 06220, Turkey; sevilay.bahadir@hotmail.com (H.S.B.); dtozlemseckin@gmail.com (Ö.S.K.)

**Keywords:** adhesive dentistry, artificially demineralized dentin, dentin-bonding agents, microshear bond strength, resin composites, selective dentin etching

## Abstract

**Highlights:**

**What are the main findings?**

- Sound dentin showed higher bond strength than artificially demineralized dentin.
- Three-second selective dentin etching showed higher immediate bond strength than self-etching after 24-h water storage.
- Resin composite type did not significantly affect microshear bond strength.
- No significant interactions were detected among the three study factors.

**What are the implications of the main findings?**

- Dentin substrate and etching strategy are key determinants of bonding performance.
- No statistically significant difference in immediate bond strength was detected between three-second selective dentin etching and etch-and-rinse.
- No statistically significant effect of resin composite type on immediate bond strength was detected.

**Abstract:**

This study evaluated the effects of dentin substrate, adhesive strategy, and resin composite type on microshear bond strength (μSBS), with particular emphasis on three-second selective dentin etching of artificially demineralized dentin (ADD). Flat midcoronal dentin surfaces from 144 human permanent third molars were allocated according to dentin substrate, adhesive strategy, and resin composite type. ADD was produced using a 14-day pH-cycling protocol. Etch-and-rinse (E&R), self-etch (SE), and three-second selective dentin etching (SDE) strategies were evaluated with a thermoviscous bulk-fill and an injectable resin composite. μSBS was tested after 24-h water storage. Data were analyzed using three-way analysis of variance and heteroscedasticity-robust HC3 tests, with Bonferroni-adjusted pairwise comparisons. Sound dentin showed higher μSBS than ADD (*p* < 0.001), and adhesive strategy had a significant effect (*p* = 0.006). SDE showed higher μSBS than SE after Bonferroni adjustment (*p* = 0.0246), whereas the E&R–SE difference did not meet the adjusted significance threshold (*p* = 0.0503). No statistically significant difference was detected between SDE and E&R. The resin composite main effect and interactions were not statistically significant. These findings concern immediate bond strength after 24-h water storage and do not establish long-term bonding performance.

## 1. Introduction

Minimally invasive dentistry aims to preserve as much healthy tooth structure as possible during caries removal. As a result, clinicians often eliminate the outer, irreversibly infected dentin while intentionally retaining the underlying caries-affected dentin, which remains partially demineralized but still capable of remineralization [1,2,3,4]. However, because caries-affected dentin differs structurally and mechanically from sound dentin (SoD), achieving a stable and durable bond to this substrate continues to be a clinical challenge [5]. Previous evidence indicates lower bond strength to caries-affected dentin than to sound dentin across different adhesive strategies, with recent systematic reviews further demonstrating that aging compromises bonding durability [6,7].

Universal adhesives have gained widespread acceptance because they can be applied using different etching strategies and simplify clinical procedures [8,9]. However, recent reviews emphasize that the long-term durability of the resin–dentin interface remains one of the major unresolved issues in adhesive dentistry, and that bonding performance can vary depending on the selected etching method, adhesive composition, and dentin substrate. This variability has been attributed to differences in adhesive composition, acidity, and interaction with dentin substrates [10]. Furthermore, universal adhesives intended for similar clinical applications may exhibit different bonding performance because of variations in functional monomers, acidity, and chemical composition [11]. Although systematic reviews and meta-analyses have reported comparable immediate dentin bond strength between the etch-and-rinse and self-etch application modes, bond durability may decline over time regardless of the strategy used [12]. Consequently, optimizing the application strategy of universal adhesives remains an important focus of contemporary adhesive research. Among the currently available application strategies, selective dentin etching (SDE), also referred to as short dentin etching, has been proposed as an intermediate approach between self-etch and etch-and-rinse protocols. This technique involves brief phosphoric acid application to dentin, producing controlled superficial demineralization while preserving residual hydroxyapatite for chemical interaction with functional adhesive monomers [13]. A recent systematic review demonstrated that short-term dentin etching protocols, particularly a 3-s etching procedure, may enhance immediate bond strength and improve hybrid layer stability by limiting excessive demineralization and preserving hydroxyapatite [14]. Likewise, experimental studies have reported improved monomer infiltration and hybrid layer formation following controlled dentin etching [13,15]. However, SDE does not consistently provide higher bond strength than conventional self-etch or etch-and-rinse approaches, and its effectiveness may vary according to the adhesive system and dentin substrate [16].

The effectiveness of selective dentin etching on caries-affected dentin is particularly uncertain because of the altered structural and chemical characteristics of this substrate. Compared with sound dentin, caries-affected dentin exhibits reduced mineral content, increased porosity, and partially denatured collagen, all of which may impair resin infiltration and compromise bond durability [3,17]. Although several studies have evaluated adhesive strategies on caries-affected dentin [18], evidence regarding selective dentin etching remains limited.

Alongside advances in adhesive strategies, contemporary resin composites have also been developed to simplify restorative procedures while improving adaptation and mechanical performance. Thermoviscous preheated bulk-fill resin composites temporarily reduce viscosity during heating, improving flow and cavity adaptation without compromising mechanical properties. Their increased translucency also facilitates deeper light penetration and efficient bulk placement [19,20,21,22,23]. A clinical trial has demonstrated favorable clinical performance and high survival rates of thermoviscous bulk-fill composite [24]. In addition, systematic review and meta-analytic evidence has shown comparable clinical performance between bulk-fill and conventional resin composites in posterior restorations [25].

In parallel, highly filled flowable materials referred to as injectable resin composites have been introduced for direct restorations, offering flowable handling and direct syringe delivery. Their use in restorative techniques has been described in a clinical case report [26], and their chemical and optical stability under wet conditions has been investigated [27].

Although contemporary resin composite technologies have demonstrated favorable physical and clinical performance, their influence on the bonding effectiveness of universal adhesives has received limited attention. Previous research has largely addressed adhesive application strategies [10,11,16] and contemporary resin composite technologies [19,20,21,22,23,24,26] separately, whereas evidence regarding their combined effects across different dentin substrates remains scarce. In particular, little is known about the bonding performance of different universal adhesive application strategies when contemporary resin composite technologies are used on artificially demineralized dentin. To the best of our knowledge, no previous study has simultaneously evaluated etch-and-rinse, self-etch, and selective dentin etching strategies in combination with thermoviscous preheated bulk-fill and injectable resin composites on both sound and artificially demineralized dentin.

Therefore, the present study aimed to evaluate the effects of dentin substrate, adhesive application strategy, and resin composite type on the microshear bond strength of a universal adhesive. Etch-and-rinse, self-etch, and three-second selective dentin etching strategies were evaluated in combination with a thermoviscous preheated bulk-fill resin composite and an injectable universal resin composite on sound dentin and artificially demineralized dentin. Sound dentin served as the experimental control substrate.

The research hypotheses were as follows:

**H1.**  *Dentin substrate would significantly influence microshear bond strength, with lower bond strength expected for artificially demineralized dentin than for sound dentin.*

**H2.**  *Adhesive application strategy would significantly influence microshear bond strength, with differences expected among the etch-and-rinse, self-etch, and selective dentin etching strategies.*

**H3.**  *Resin composite type would significantly influence microshear bond strength, with differences expected between the thermoviscous preheated bulk-fill resin composite and the injectable universal resin composite*.

**H4.**  *Significant two-way and three-way interactions would occur among dentin substrate, adhesive application strategy, and resin composite type in their effects on microshear bond strength*.

## 2. Materials and Methods

### 2.1. Sample Size Calculation and Ethical Approval

Ethical approval for this study was obtained from the Ethics Committee of Ankara Yildirim Beyazit University (Approval No. 2024-09/839). Sample size was calculated a priori using G*Power software (version 3.1; Heinrich Heine University, Düsseldorf, Germany). Written informed consent for the use of extracted teeth in this study was obtained from the donors. Under the “F tests” family, “ANOVA: Fixed effects, omnibus, one-way” was selected for the 12 experimental groups in the 2 × 2 × 3 design. An effect size of f = 0.40, corresponding to a large effect according to Cohen’s convention, was assumed. The significance level was set at α = 0.05 and the target power at 0.90. The calculation was based on the overall difference among the 12 groups rather than on a specific main effect or interaction. The required total sample size was 144 teeth (12 per group; numerator degrees of freedom = 11; denominator degrees of freedom = 132), with an achieved power of 0.9036.

A total of 144 sound human permanent third molars, extracted for clinical reasons because they were nonfunctional, were included after eligibility screening. Before specimen preparation, the teeth were examined under a stereomicroscope; teeth with visible caries, restorations, cracks, or developmental defects were excluded. After extraction, the teeth were cleaned with a curette and disinfected with 0.1% thymol solution. Then the teeth were transferred to distilled water and stored at 4 °C for no longer than six months before use. All teeth were embedded in cylindrical silicone molds using autopolymerizing acrylic resin (Imcryl, Konya, Turkey). Under running water, a low-speed diamond separator (Microcut 125, Metkon, Bursa, Turkey) was used to remove the occlusal third and prepare the mid-coronal dentin for flat surfaces. The dentin surfaces were prepared using 1200-grit SiC paper to produce a standardized smear layer. After enamel removal and preparation of the flat dentin surfaces, the teeth were randomly allocated to the experimental groups by drawing lots before the pH-cycling procedure. The following techniques were applied to the two groups of teeth: Group 1: control (SoD) and Group 2: artificially demineralized dentin (ADD) produced using a standardized pH-cycling protocol.

### 2.2. pH-Cycling Protocol for Artificially Demineralized Dentin

Artificially demineralized dentin (ADD) was prepared using a pH-cycling approach based on a previously reported protocol for human mid-coronal dentin [28]. Each specimen was first immersed for 8 h in 10 mL of demineralizing solution (2.2 mM CaCl_2_, 2.2 mM NaH_2_PO_4_, and 50 mM acetic acid; pH adjusted to 4.8) and then for 16 h in 10 mL of remineralizing solution (1.5 mM CaCl_2_, 0.9 mM NaH_2_PO_4_, and 0.15 M KCl; pH adjusted to 7.0). The pH-cycling protocol was repeated daily for 14 consecutive days at room temperature without agitation. The demineralizing and remineralizing solutions were renewed after each cycle. The substrate was not characterized by microhardness, lesion depth, mineral content, or microscopic analyses after pH cycling; therefore, the extent and uniformity of demineralization could not be established.

### 2.3. Bonding Procedures

A universal adhesive (FuturaBond U, VOCO, Cuxhaven, Germany) was applied using the etch-and-rinse and self-etch procedures described in the manufacturer’s instructions. Three-second selective dentin etching was evaluated as an experimental modification of the etch-and-rinse procedure (Table 1).

The same 35% phosphoric acid and application method were used for the etch-and-rinse and selective dentin etching groups; only the etching duration differed. In both groups, after rinsing and before adhesive application, excess water was removed by gently air-drying the dentin for 5 s with the same air–water syringe, held at a consistent distance and pressure across specimens.

For the etch-and-rinse strategy, the dentin surfaces were etched with 35% phosphoric acid for 15 s and rinsed with water for approximately 20 s. The adhesive was actively applied using a microbrush for 20 s, air-thinned for at least 5 s, and light-cured for 10 s.

For the self-etch strategy, the adhesive was applied directly to the dentin surfaces without prior phosphoric acid etching, actively applied for 20 s, air-thinned for at least 5 s, and light-cured for 10 s.

For the selective dentin etching strategy, the dentin surfaces were etched with 35% phosphoric acid for 3 s and rinsed with water for approximately 20 s. The three-second interval was measured using a digital stopwatch, starting when the acid first contacted the dentin. The adhesive was then actively applied for 20 s, air-thinned for at least 5 s, and light-cured for 10 s.

The adhesive was light-cured using an LED light-curing unit (Elipar S10, 3M ESPE, St. Paul, MN, USA) with an irradiance of approximately 1200 mW/cm^2^ and a wavelength range of 430–480 nm. The irradiance was verified using the radiometer integrated into the manufacturer’s charging unit before each bonding procedure.

Each adhesive subgroup was further divided according to resin composite type (*n* = 12): a thermoviscous bulk-fill resin composite (VisCalor Bulk, VOCO, Cuxhaven, Germany) or an injectable universal resin composite (G-ænial Universal Injectable, GC, Tokyo, Japan). A Tygon tube (0.8-mm internal diameter and 1-mm height) was positioned on the adhesive-treated dentin surface, and the assigned resin composite was inserted into the tube. Only one composite cylinder was bonded to each tooth. Thus, each tooth contributed one μSBS measurement and constituted one independent experimental unit. VisCalor Bulk was preheated at 65 °C for 30 s using the VisCalor Dispenser (VOCO, Cuxhaven, Germany), with a working time of 2 min and 30 s according to the manufacturer’s instructions. Both resin composites were light-cured for 10 s according to the manufacturer’s instructions (Table 1). The light-curing tip was positioned as close as possible to the composite surface. Specimens were stored in distilled water at 37 °C for 24 h, after which the Tygon tubes were removed using a sharp blade. All specimen preparation procedures were performed by a single operator.

### 2.4. Microshear Bond Strength (μSBS) Evaluation

**Figure 1:**
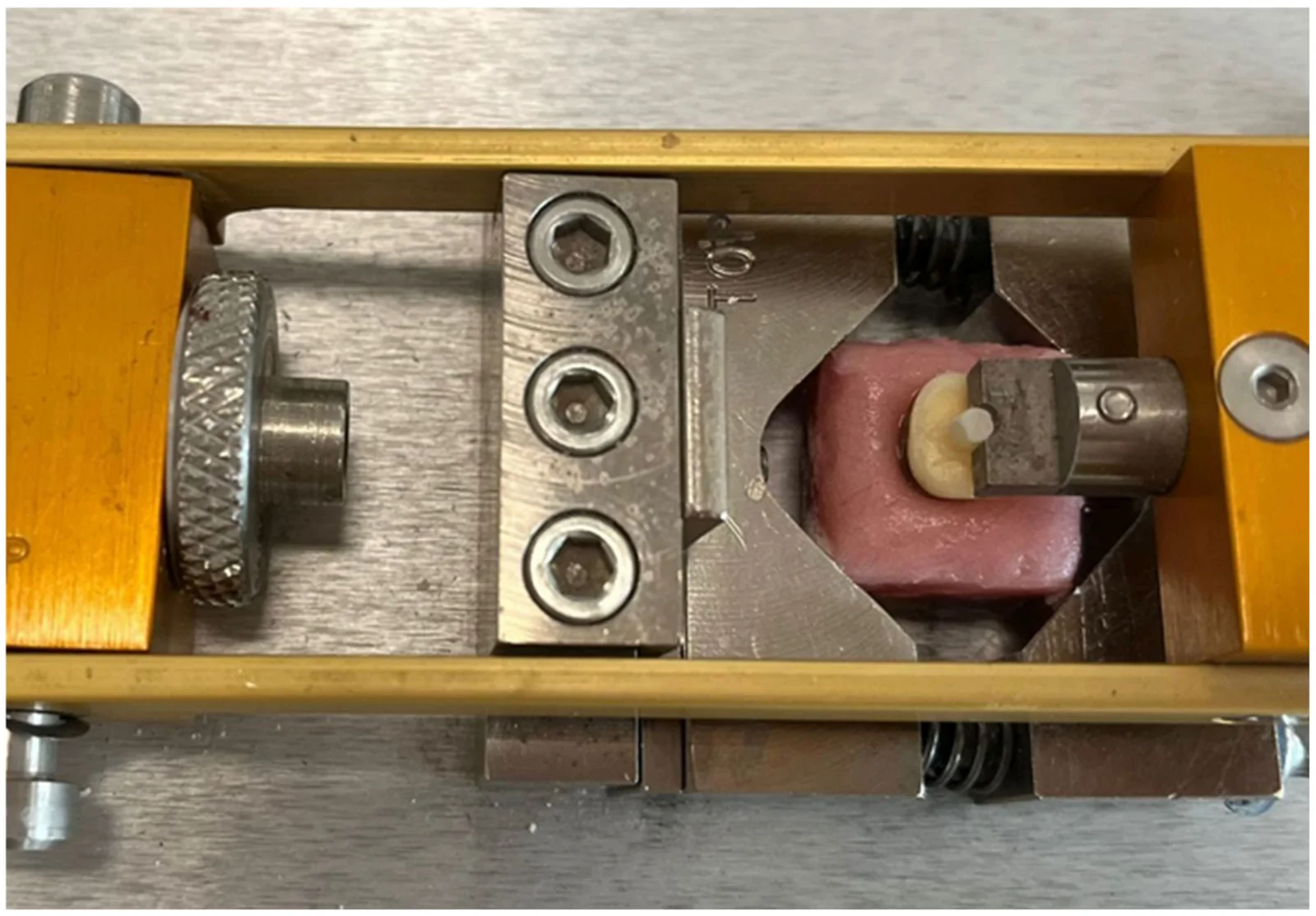
Specimen positioned in the universal testing machine for microshear bond strength testing.

Specimens were subjected to μSBS testing using a universal testing machine (Z010, Zwick, Ulm, Germany) at a crosshead speed of 1.0 mm/min until failure. A 0.4-mm-thick blade was positioned adjacent to the resin–dentin interface (Figure 1). The maximum load at failure (N) was recorded and divided by the bonded area (mm^2^) to calculate μSBS in megapascals (MPa). The bonded area was calculated based on the internal diameter of the Tygon tube (0.8 mm). No premature debonding occurred, and no specimens were excluded; therefore, all 144 specimens (*n* = 12 per experimental subgroup) were included in the μSBS testing and statistical analysis. The operator performing the μSBS test was blinded to group allocation.

### 2.5. Failure Mode Assessment

After μSBS testing, failure modes were examined under a stereomicroscope (Euromax, Hamburg, Germany) at 20× magnification and categorized as: Type I—adhesive failure (at the resin–dentin interface), Type II—cohesive failure (within dentin or resin composite), and Type III—mixed failure (combination of adhesive and cohesive components). Failure modes were assessed by an examiner who was blinded to group allocation.

### 2.6. Statistical Analysis

Statistical analyses were performed using the Statistical Package for the Social Sciences (SPSS, version 25.0; IBM Corp., Armonk, NY, USA). The heteroscedasticity-robust HC3 Wald tests and HC3-based pairwise comparisons were performed in Python (version 3.12.14) using NumPy (version 2.3.5) and SciPy (version 1.17.0). Data normality was assessed using the Shapiro–Wilk test, and the homogeneity of variances was evaluated using Levene’s test. Levene’s test indicated variance heterogeneity among the groups (*p* < 0.001). Each of the 12 groups in the study’s 2 × 2 × 3 factorial design comprised 12 independent observations (total *n* = 144). Results from a standard three-way ANOVA were reported; however, since equal group sizes do not entirely eliminate the potential effects of variance heterogeneity, heteroskedasticity-robust HC3 Wald tests were prioritized for interpreting the findings. Residual-versus-predicted value plots and Q–Q plots were examined, and potentially influential observations were assessed using leverage values and Cook’s distance. The *p*-values for the three pairwise comparisons between adhesive strategies were adjusted using the Bonferroni method, and the corresponding 98.33% confidence intervals were calculated. Descriptive results are presented as mean ± standard deviation (SD). Statistical significance was set at *p* < 0.05. Failure mode distributions were summarized using descriptive statistics. The reporting of this in vitro study was informed by the transparency and methodological considerations proposed in the CRIS concept note [29].

## 3. Results

### 3.1. Microshear Bond Strength

**Figure 2:**
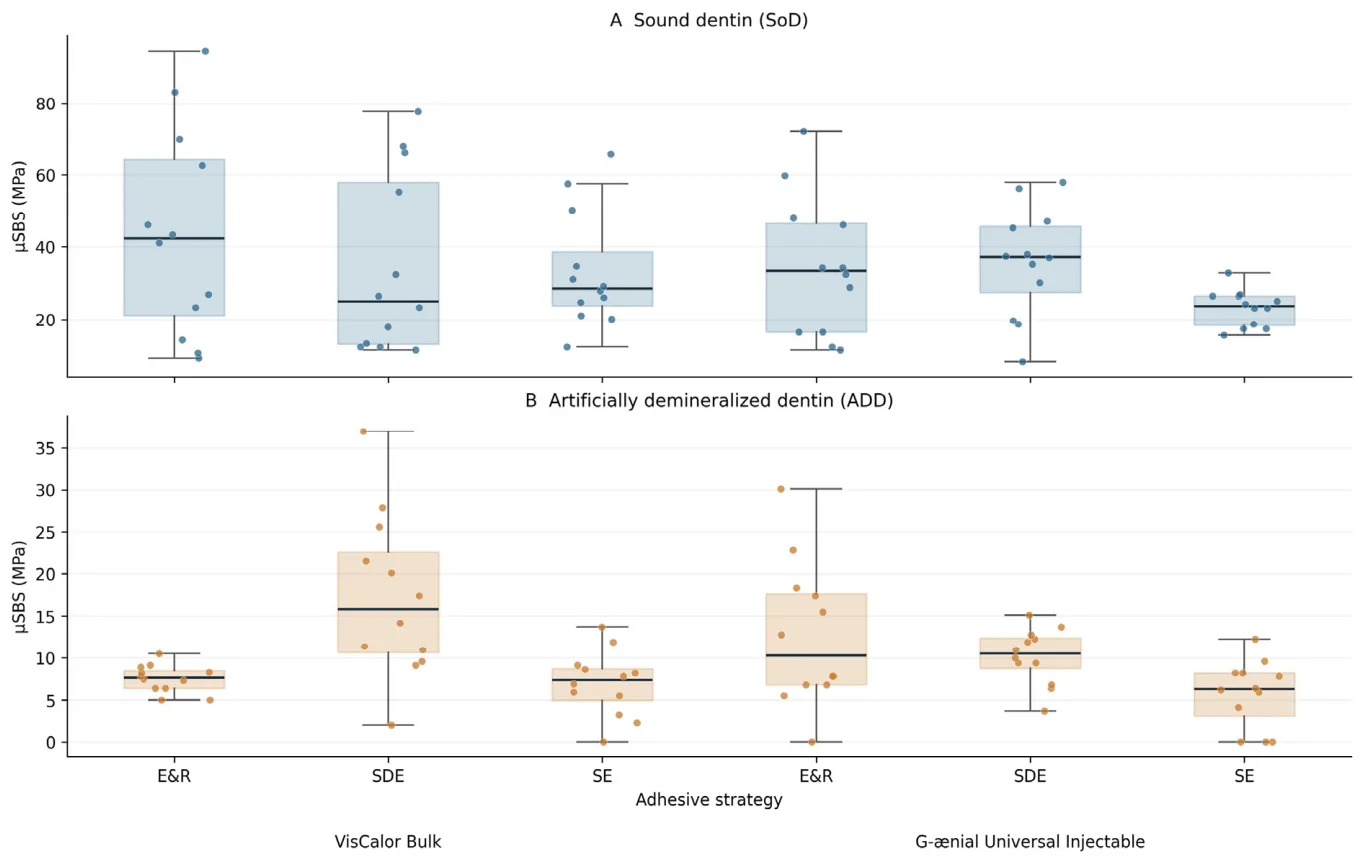
Microshear bond strength (μSBS) values in the 12 experimental groups. Boxplots show the median and interquartile range; overlaid points represent individual teeth (*n* = 12 per group). SoD, sound dentin; ADD, artificially demineralized dentin; E&R, etch-and-rinse; SE, self-etch; SDE, selective dentin etching.

Mean ± standard deviation values for all experimental groups are presented in Table 2. The conventional three-way ANOVA results are presented in Table 3. In the HC3-robust analysis, dentin substrate (*p* < 0.001) and adhesive strategy (*p* = 0.006) had significant main effects on μSBS (Table 4). Sound dentin exhibited higher μSBS than artificially demineralized dentin. After Bonferroni adjustment, SDE showed higher μSBS than SE (mean difference, 7.22 MPa; adjusted *p* = 0.0246). The estimated E&R–SE difference was 7.28 MPa but did not meet the adjusted significance threshold (*p* = 0.0503). No statistically significant difference was detected between E&R and SDE (*p* = 1.000) (Table 5). The resin composite main effect and all interaction effects were not statistically significant. HC3-robust effect estimates and 95% confidence intervals for the interaction contrasts are provided in Supplementary Table S1. The distributions of μSBS values and individual observations across all 12 experimental groups are shown in Figure 2. Model diagnostic plots are provided in Supplementary Figure S1.

### 3.2. Failure Mode Analysis

**Figure 3:**
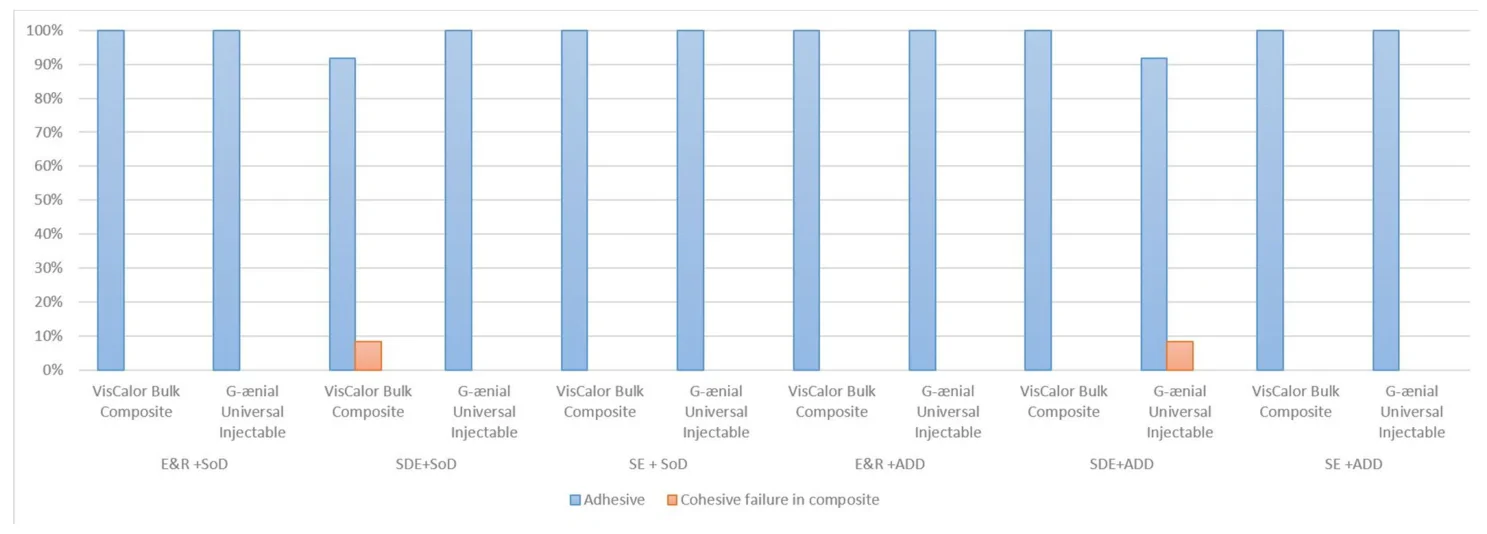
Distribution of failure modes according to dentin substrate, adhesive strategy, and resin composite type. Both cohesive failures occurred within the resin composite; no cohesive failures within dentin were observed.

Failure mode analysis revealed that adhesive failure predominated across all experimental groups. Cohesive failures within the resin composite occurred in one specimen in the SoD/SDE/VisCalor Bulk group (1/12; 8.3%) and one specimen in the ADD/SDE/G-ænial Universal Injectable group (1/12; 8.3%). No mixed failures were observed (Figure 3).

## 4. Discussion

The present findings highlight the importance of dentin substrate in the immediate bonding performance of the universal adhesive. The first research hypothesis was supported, as dentin substrate significantly influenced μSBS, with SoD exhibiting significantly higher values than ADD (*p* < 0.001). This finding is consistent with previous studies [18,30] and a systematic review and meta-analysis reporting lower bond strength to caries-affected dentin than to sound dentin [17]. Naturally occurring caries-affected dentin is characterized by reduced mineral content, increased porosity, and alterations in the collagen-rich organic matrix, which may compromise adhesive infiltration and hybrid layer formation [30,31]. However, these structural characteristics were not directly evaluated in the artificially induced substrate used in the present study. Therefore, the lower μSBS observed for ADD should be interpreted in the context of the experimentally altered dentin substrate without attributing it to specific structural or biological changes that were not assessed.

The second research hypothesis was partially supported because adhesive application strategy significantly influenced μSBS. In the HC3-robust pairwise analysis, SDE showed higher immediate μSBS than SE, whereas the E&R–SE difference did not reach statistical significance after Bonferroni adjustment. This difference may be related to the effects of phosphoric acid conditioning on the dentin surface and subsequent adhesive infiltration. Previous studies have similarly demonstrated that bonding effectiveness is influenced by both dentin substrate characteristics and adhesive approach [18,30,32]. Microscopic observations of naturally occurring caries-affected dentin have shown partial demineralization, a sparser distribution of hydroxyapatite, and altered surface morphology following acid etching compared with normal dentin [31]. These substrate characteristics may modify the response to phosphoric acid conditioning and subsequent adhesive infiltration [17,30]. In E&R protocols, phosphoric acid produces extensive dentin demineralization, and in an already demineralized substrate, the depth of acid penetration may exceed the depth of resin monomer infiltration, potentially compromising hybrid layer integrity [17,30]. However, these mechanisms have primarily been described for naturally occurring caries-affected dentin and were not directly evaluated in the artificially induced substrate used in the present study. Therefore, they should be considered possible explanations rather than direct mechanisms underlying the present findings.

Three-second SDE showed significantly higher immediate μSBS than SE, while no statistically significant difference was detected between SDE and E&R. The latter finding should not be interpreted as evidence that the two strategies are equivalent. Short-duration dentin etching has been reported to limit excessive demineralization while preserving residual hydroxyapatite and facilitating resin infiltration [14]. Residual hydroxyapatite may contribute to adhesive interface stability through chemical interaction with functional monomers such as 10-MDP [33]. These mechanisms were not directly assessed in the present study. The dentin substrate × adhesive strategy interaction was not statistically significant in the HC3-robust analysis, providing no statistical evidence that the relative performance of the adhesive strategies differed between SoD and ADD under the experimental conditions tested. Variations in dentin depth during specimen preparation may also contribute to variability in μSBS. Hosseini et al. highlighted the difficulty of ensuring a homogeneous dentin depth within horizontal sections, emphasizing the importance of depth standardization in laboratory bonding studies [34]. These immediate findings do not establish the long-term effectiveness of SDE. Marchesi et al. reported no significant differences in baseline bond strength among the tested groups, but differences emerged after one year of storage, highlighting the importance of aging when evaluating adhesive performance [35].

Resin composite type was included as an experimental factor because differences in viscosity during placement, adaptation to the bonded surface, and polymerization behavior could potentially affect stress transfer at the adhesive interface and, consequently, the measured μSBS. Preheating VisCalor Bulk was expected to temporarily reduce its viscosity, while G-ænial Universal Injectable was placed by direct injection. These proposed mechanisms were not directly measured in the present study. The hypothesis that resin composite type would influence μSBS was not supported, as no statistically significant difference was detected between the composites under the conditions tested. The potential advantages of thermoviscosity, bulk placement, cavity adaptation, and depth of cure are primarily relevant to larger restorative volumes and clinically shaped cavities. Here, both composites were tested as cylinders only 0.8 mm in diameter and 1 mm in height, and failures occurred predominantly at the adhesive interface. This test configuration may have limited the ability to detect material-related differences relevant to clinical restorations. Therefore, the findings should not be interpreted as evidence of clinical equivalence or used to guide composite selection.

The fourth research hypothesis was not supported, as no significant interactions were detected among dentin substrate, adhesive application strategy, and resin composite type. The absence of a dentin substrate × adhesive strategy interaction indicates that the influence of adhesive strategy did not significantly differ between SoD and ADD under the conditions evaluated. Similarly, the absence of interactions involving the resin composite type indicates that the effects of dentin substrate and adhesive strategy were not significantly modified by the composite used. Previous evidence has emphasized that the bonding performance of universal adhesives may be influenced by their chemical composition and application approach [10,11] and that the response to different etching modes may vary among adhesive formulations [16]. In the present study, however, no statistical evidence was found for a substrate- or composite-dependent response to the adhesive strategies evaluated. The nonsignificant interaction tests do not establish that the effects were identical across dentin substrates or resin composites; the confidence intervals for the interaction contrasts indicate the range of effects compatible with the data.

Failure mode analysis revealed predominantly adhesive failures across the experimental groups, whereas cohesive failures were infrequently observed. Previous evidence has shown considerable variability in failure patterns between sound and caries-affected dentin, with most studies reporting no appreciable differences between substrates [17]. The predominance of adhesive failures observed in the present study is therefore consistent with the heterogeneous failure patterns reported in previous dentin bond strength studies.

This study has several limitations that should be considered when interpreting the findings. First, artificially demineralized dentin has been used as an experimental substrate in previous dentin-bonding studies [6]. However, the pH-cycled dentin in the present study was not characterized by microhardness testing, lesion-depth measurement, mineral analysis, or microscopy. Therefore, the extent and uniformity of demineralization could not be verified. This experimental substrate cannot be assumed to fully reproduce naturally occurring caries-affected dentin, and the findings should not be directly generalized to clinical carious lesions. Second, bond strength was evaluated after 24 h of water storage without thermal or mechanical aging; consequently, the results reflect immediate bonding performance and provide no information regarding the long-term durability of the adhesive interface. The use of a single universal adhesive also limits the generalizability of the findings to adhesives with different compositions and functional monomers. In addition, the absence of a conventional resin composite control restricts the interpretation of the performance of the two contemporary resin composite technologies relative to conventional restorative materials. The small dimensions of the bonded composite cylinders inherent to the microshear bond strength test do not reproduce clinical conditions in which the potential advantages of thermoviscous bulk placement, such as improved flow, cavity adaptation, and depth of cure, would be expected to become relevant. Furthermore, although the thermoviscous bulk-fill resin composite was preheated according to the manufacturer’s instructions, its actual temperature at the time of placement was not measured. Finally, the absence of microscopic analysis of the adhesive interface precluded direct evaluation of the micromorphological effects associated with the different adhesive strategies.

Future studies should incorporate characterization of the artificially demineralized dentin model, naturally occurring caries-affected dentin, thermal and mechanical aging, different universal adhesive formulations, conventional resin composite controls, and interfacial analyses to determine whether the immediate bonding patterns observed in the present study are maintained under more clinically relevant conditions.

## 5. Conclusions

Within the limitations of this in vitro study, dentin substrate and adhesive application strategy significantly influenced immediate microshear bond strength after 24 h of water storage, whereas no statistically significant effect of resin composite type was detected. Sound dentin showed higher μSBS than artificially demineralized dentin. Three-second selective dentin etching produced higher immediate μSBS than self-etching, whereas the E&R–SE difference did not remain statistically significant after robust analysis and adjustment for multiple comparisons. These findings are limited to immediate bond strength under the experimental conditions tested and do not establish the long-term performance of the adhesive strategies or the clinical equivalence of the resin composites.

## Figures and Tables

**Table 1:** Materials used in the study, manufacturers, main components, and application procedures.

Material (Lot)	Manufacturer	Main Components	Application Protocol
VisCalor Bulk—thermoviscous bulk-fill resin composite Lot: 2344303	VOCO, Cuxhaven, Germany	Matrix: Bis-GMA, TCDDMA, initiators, stabilisers, colour pigments Filler: Barium aluminium borosilicate and silicon dioxide Content by weight: 83%	Preheated in the VisCalor Dispenser at 65 °C for 30 s (setting 1), kept warm for up to 2 min 30 s, inserted into the Tygon tube, and light-cured for 10 s.
G-aenial Universal Injectable Universal light-cure radiopaque injectable nanocomposite Lot: 2306091	GC, Tokyo, Japan	Matrix: Bis-MEPP Filler: Silicon dioxide, Barium glass Filler Content by weight: 69 wt% Barium glass filler particle size: approximately 150 nm	The composite was inserted into the Tygon tube and light-cured for 10 s according to the manufacturer’s instructions.
FuturaBond U Universal Bond Lot: 2350596	VOCO, Cuxhaven, Germany	Bisphenol A diglycidyl methacrylate, 2-hydroxyethyl methacrylate, 1,6-hexanediyl bismethacrylate, acidic adhesive monomer, urethane dimethacrylate, catalyst, initiators, stabilisers	Etch-and-Rinse strategy: The dentin surface was etched with 35% phosphoric acid for 15 s, rinsed with water for approximately 20 s, and gently air-dried. The adhesive was then actively rubbed for 20 s, air-thinned for at least 5 s, and light-cured for 10 s. Self-Etch strategy: The adhesive was applied directly to the dentin surface without phosphoric acid etching, actively rubbed for 20 s, air-thinned for at least 5 s, and light-cured for 10 s. Selective Dentin Etching strategy: The dentin surface was etched with 35% phosphoric acid for 3 s, rinsed with water for approximately 20 s, and gently air-dried for 5 s. The adhesive was then actively rubbed for 20 s, air-thinned for at least 5 s, and light-cured for 10 s.

**Table 2:** Mean ± Standard Deviation of Microshear Bond Strength (μSBS) Values for the Experimental Groups (MPa).

	Artificially Demineralized Dentin (ADD)			Sound Dentin (SoD)		
	E&R Mean ± SD	SE Mean ± SD	SDE Mean ± SD	E&R Mean ± SD	SE Mean ± SD	SDE Mean ± SD
VisCalor Bulk composite	7.5 ± 1.6	6.9 ± 3.8	17.2 ± 9.7	43.7 ± 28.6	33.3 ± 16.1	34.6 ± 24.9
G-aenial Universal Injectable resin composite	12.6 ± 8.5	5.7 ± 4.0	10.2 ± 3.3	34.3 ± 19.2	23.1 ± 5.0	35.8 ± 15.0

Values are presented as mean ± standard deviation (MPa); *n* = 12 per experimental group.

**Table 3:** Conventional Three-Way ANOVA for the Effects of Dentin Substrate, Adhesive Strategy, Resin Composite Type, and Their Interactions on Microshear Bond Strength.

Source	Type III SS	df	MS	F	*p*-Value	Partial η^2^
Corrected model	24,713.596	11	2246.691	10.602	<0.001	0.469
Dentin substrate	20,986.351	1	20,986.351	99.036	<0.001	0.429
Resin composite type	465.840	1	465.840	2.198	0.141	0.016
Adhesive strategy	1,681.115	2	840.558	3.967	0.021	0.057
Dentin substrate × Resin composite type	235.111	1	235.111	1.110	0.294	0.008
Dentin substrate × Adhesive strategy	417.154	2	208.577	0.984	0.376	0.015
Resin composite type × Adhesive strategy	85.815	2	42.908	0.202	0.817	0.003
Dentin substrate × Resin composite type × Adhesive strategy	842.209	2	421.105	1.987	0.141	0.029
Error	27,971.690	132	211.907	—	—	—
Corrected total	52,685.286	143	—	—	—	—

Abbreviations: SS, sum of squares; df, degrees of freedom; MS, mean square; partial η^2^, partial eta squared. Model fit: R^2^ = 0.469; adjusted R^2^ = 0.425. Statistical significance was set at *p* < 0.05.

**Table 4:** Heteroscedasticity-robust HC3 Wald tests for the factorial effects on microshear bond strength.

Effect	df_1_	df_2_	HC3 Wald F	*p*-Value
Dentin substrate	1	132	90.783	<0.001
Resin composite type	1	132	2.015	0.158
Adhesive strategy	2	132	5.350	0.006
Dentin substrate × Resin composite type	1	132	1.017	0.315
Dentin substrate × Adhesive strategy	2	132	0.743	0.478
Resin composite type × Adhesive strategy	2	132	0.259	0.772
Dentin substrate × Resin composite type × Adhesive strategy	2	132	1.634	0.199

HC3, heteroscedasticity-consistent covariance estimator. All tests used the same 2 × 2 × 3 factorial model. df_1_, numerator degrees of freedom; df_2_, denominator degrees of freedom.

**Table 5:** HC3-robust pairwise comparisons between adhesive strategies.

Comparison	Estimated Mean Difference (MPa)	Classical Bonferroni-Adjusted *p*	HC3 Bonferroni-Adjusted *p*	98.33% HC3 Confidence Interval (MPa)
E&R-SE	7.277	0.047	0.0503	−0.01 to 14.56
SDE-SE	7.219	0.049	0.0246	0.70 to 13.74
E&R-SDE	0.058	1.000	1.000	−8.56 to 8.68

Mean differences are averaged across dentin substrates and resin composite types. HC3 confidence intervals and *p* values are adjusted for the three pairwise comparisons using the Bonferroni method.

## Data Availability

The raw data supporting the conclusions of this article will be made available by the authors on request.

## Supplementary Materials

The following supporting information can be downloaded at: https://www.mdpi.com/article/10.3390/ma19194227/s1, Figure S1: Diagnostic plots for the three-way factorial model: residuals versus fitted values, normal Q–Q plot of model residuals, and Cook’s distance for individual observations. The dashed horizontal line in the Cook’s distance panel indicates the screening threshold of 4/n; Table S1: HC3-robust estimates and 95% confidence intervals for interaction contrasts in immediate microshear bond strength.

## Abbreviations

The following abbreviations are used in this manuscript:

μSBS	Microshear bond strength
E&R	Etch-and-rinse
SE	Self-etch
SDE	Selective dentin etching
ADD	Artificially demineralized dentin
SoD	Sound dentin
Bis-GMA	Bisphenol A glycidyl methacrylate
UDMA	Urethane dimethacrylate
HEMA	2-hydroxyethyl methacrylate
Bis-MEPP	2, 2-bis(4-methacryloxypolyethoxyphenyl)propane
