# Supplementary material for: Universal Adhesive Bonding to Sound and Artificially Demineralized Dentin: The Role of Etching Strategy and Resin Composite Type
Source: Materials (Basel). 2026 Oct 5;19(19):4227. doi: 10.3390/ma19194227 (PMC13666021; doi:10.3390/ma19194227)
Supplement: Supplementary file 1 [file materials-19-04227-s001.zip › materials-4585095-supplementary.pdf]

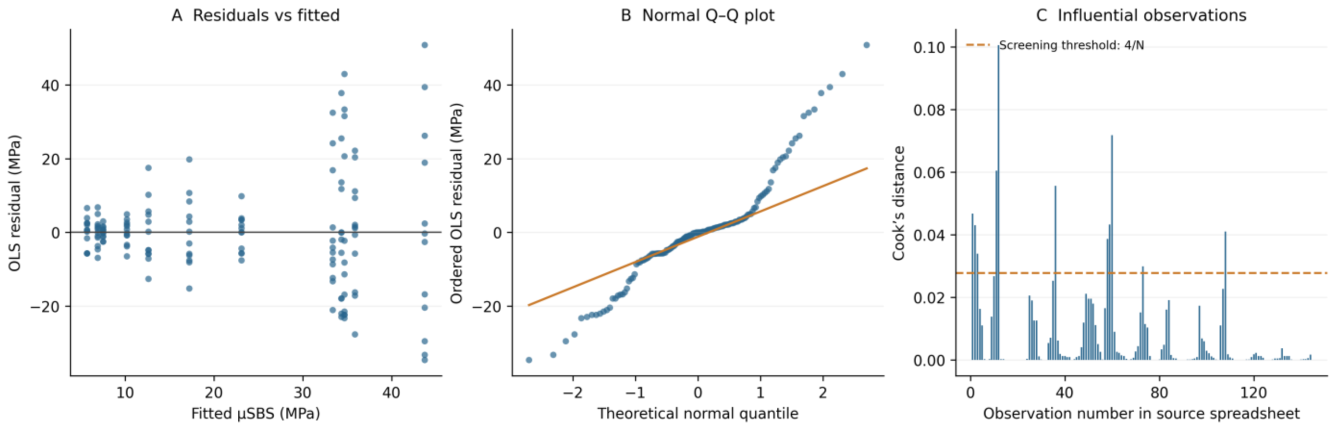

Supplementary Figure S1: Diagnostic plots for the three-way factorial model: residuals versus fitted values, normal Q–Q plot of model residuals, and Cook's distance for individual observations. The dashed horizontal line in the Cook's distance panel indicates the screening threshold of 4/n.

Supplementary Table S1: HC3-robust estimates and 95% confidence intervals for interaction contrasts in immediate microshear bond strength

Interaction contrast	Estimated difference in differences (MPa)	95% HC3 confidence interval (MPa)
**Dentin substrate × resin composite type**	5.11	−4.91 to 15.14
**Dentin substrate × E&R–SE**	7.04	−4.85 to 18.92
**Dentin substrate × SDE–SE**	−0.35	−10.99 to 10.29
**Resin composite type × E&R–SE**	−3.60	−15.48 to 8.29
**Resin composite type × SDE–SE**	−2.81	−13.45 to 7.83
**Dentin substrate × resin composite type × E&R–SE**	5.41	−18.36 to 29.18
**Dentin substrate × resin composite type × SDE–SE**	−17.28	−38.55 to 4.00

Estimates are differences between group contrasts, based on the 2 × 2 × 3 factorial model. Dentin substrate contrasts are calculated as sound dentin minus artificially demineralized dentin; resin composite contrasts are calculated as VisCalor Bulk minus G-ænial Universal Injectable. Effects are averaged equally across any factor not included in the contrast. Confidence intervals are individual, unadjusted 95% HC3 intervals. Their inclusion of zero does not establish equivalence between groups.
